# Adherence to rehabilitation exercise and influencing factors among people with acute stroke: a cross-sectional study

**DOI:** 10.3389/fneur.2025.1554949

**Published:** 2025-02-27

**Authors:** Feng Xing, Juan Liu, Chang Mei, Jingnan Chen, Yi Wen, Jianrong Zhou, Shiqi Xie

**Affiliations:** ^1^Department of Nursing, the First Affiliated Hospital of Chongqing Medical University, Chongqing, China; ^2^Department of Neurology, the First Affiliated Hospital of Chongqing Medical University, Chongqing, China; ^3^School of Nursing, Chongqing Medical University, Chongqing, China

**Keywords:** stroke, exercise, psychological well-being, adherence, influence factor

## Abstract

**Background:**

Post-stroke rehabilitation exercise is recognized as the most effective and preferred intervention to reduce disability rates in patients. However, adherence to exercise is low among stroke patients. Previous research has mainly focused on the negative psychological factors influencing adherence, while the positive psychological aspects remain underexplored. Therefore, this study aims to investigate the factors influencing adherence to rehabilitation exercises in acute stroke patients, especially the positive psychological factors, in order to reduce the disability rate of stroke.

**Methods:**

From October 2023 to March 2024, a total of 227 patients with acute stroke were selected from a Grade-A hospital in Chongqing, China, using total sampling method. The patients’ general demographic data, Stroke Functional Exercise Adherence Questionnaire Scale (EAQ), Hospital Anxiety and Depression Scale (HAD), and Index of Subjective Well-Being Scale (IWB) were assessed. ANOVA, *t*-test analysis, correlation analysis, and multiple linear regression models were used to explore the influencing factors in people with acute stroke.

**Results:**

The total score for rehabilitation exercise adherence in acute stroke patients was 38.41 ± 11.13, corresponding to a mean adherence rate of 68.6%. Factors influencing adherence to rehabilitation exercise in acute stroke patients were identified, including age (*p* < 0.0001), presence of ICU stay (*p* = 0.03), National Institutes of Health Stroke Scale (NIHSS) score (*p* < 0.0001), and subjective well-being (*p* < 0.01).

**Conclusion:**

Adherence to rehabilitation exercises in acute stroke patients was found to be medium. Our findings highlight that age, presence of ICU stay, and NIHSS score were found to have negative correlations with adherence. While a positive correlation was observed with subjective well-being.

## Introduction

Acute stroke, frequently referred to as a cerebrovascular accident, is an acute episode of focal neurological dysfunction that persists for more than 24 h (1). Worldwide, in 2019, there were 12.2 million incident cases of stroke, 101 million prevalent cases of stroke, stroke has become the second leading cause of mortality, and the third-leading cause of death and disability combined (2). The prevalence of stroke in the young is increasing globally (3). China leads the world with the highest lifetime risk of stroke, estimated at 39.3% from the age of 25 years (4). Stroke brings great burden on individuals, families and society. In China, it estimated that the medical cost of hospitalization for stroke in 2019 was CNY 54.8 billion, of which the patient paid approximately CNY 18.3 billion (33.4%) (5).

Motor dysfunction occurs in about 69 to 80% of patients after a stroke (6). Early rehabilitation can significantly enhance clinical outcomes for individuals with an acute stroke, Patients with high adherence have better recovery of limb function and better quality of life (7). Evidence-based medicine has shown that stroke rehabilitation is the most effective approach to reducing disability in patients, with rehabilitation exercises being the preferred form of therapy for stroke survivors (8). Rehabilitation exercise adherence refers to the extent to which patients follow their prescribed exercise program (9). However, adherence to rehabilitation exercises is generally low in stroke patients. Studies have shown that during hospitalization, 63 to 82% of stroke patients adhere well to their exercises, but this rate drops to 47.41% after discharge (10). Although patients may adhere well initially, their long-term adherence tends to be poor, which has a significant impact on the rehabilitation process and treatment outcomes (11). Most studies investigating the factors influencing adherence to stroke rehabilitation exercises have focused primarily on negative psychological constructs. The prevalence of anxiety and depressive symptoms after stroke can be as high as 79%, although most estimates are about 30% (12, 13). Researches suggest that anxiety and depression have a significant impact on stroke patients’ adherence to rehabilitation exercises (14, 15). For example, the result found a direct correlation between the severity of depression and low adherence to active exercise (16). These emotional challenges have a direct impact on the functioning and quality of life of stroke survivors. With the development of positive psychology, in cardiovascular disease studies, positive psychological constructs can enhance adherence to health-promoting behaviors and promote health outcomes (17, 18); Celano et al. (19) reported psychological well-being may play an important role in adherence and cardiovascular health in patients with heart failure; Huffman et al. (20) reported the links between positive psychological well-being and greater physical activity in patients with Type 2 diabetes. The meta-integration of qualitative research on mental experience of stroke patients systematically interprets the existence of positive mental states in stroke patients after illness. However, the effect of positive psychological emotions on adherence to rehabilitation exercises in stroke patients is not known.

Therefore, based on positive and negative psychological dimensions, this study conducted a cross-sectional investigation of factors affecting adherence to rehabilitation exercises in patients with acute stroke, explored the current status and influential factors. The research findings will provide new ideas and methods for improving exercise adherence in stroke patients.

## Methods

### Design and setting

**Figure 1:**
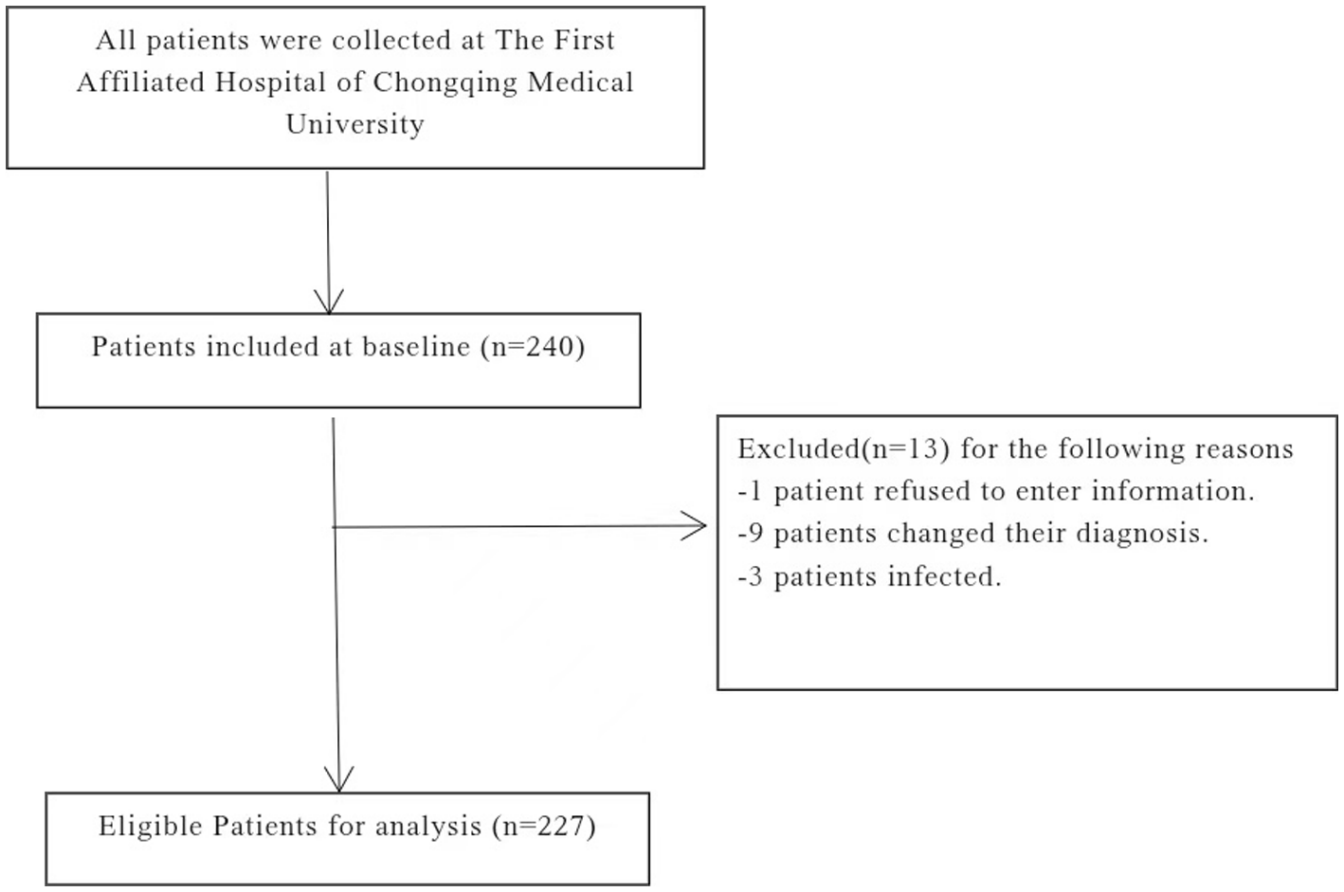
Participate in patient screening process description.

This cross-sectional study was conducted among stroke patients from October 2023 to March 2024 at a Grade-A hospital in Chongqing, China. Initially, a total of 240 patients were recruited. Subsequently, 13 individuals were excluded, resulting in 227 patients being included in the final data analysis (Figure 1).

### Participants

Using the total sampling method, a study was conducted on a sample of early stroke patients admitted to the Department of Neurology of the First Affiliated Hospital of Chongqing Medical University in Chongqing, China. Participants were required to meet the study’s inclusion and exclusion criteria. Inclusion criteria: (1) met the diagnostic criteria for acute stroke with a first-onset; (2) were aged 18 years and above; (3) exhibited muscle strength below grade 5; (4) were able to comprehend and cooperate effectively, cognitive or understanding ability has been grossly screened; (5) had received instruction in rehabilitation training; (6) willingly agreed to participate and signed the informed consent form. Exclusion criteria: (1) concomitant major diseases; (2) severe speech disorders; (3) active infections; (4) involvement in other concurrent research studies.

The study included 19 explanatory variables (15 demographic variables, rehabilitation exercise adherence, anxiety, depression, and subjective well-being).

### Measures

Four questionnaires were used in the study to collect general information, evaluate adherence to rehabilitation exercises, subjective well-being, and anxiety and depression in stroke patients.

#### General information questionnaire

A self-designed general information questionnaire was developed by reviewing relevant literature, covering demographic and sociological details such as gender, age, social support system, health insurance, educational level, monthly income, and marital status. Some of the data in our study were referenced from medical records, including NICU transfer status, dysarthria, modified Rankin Scale (mRS), National Institutes of Health Stroke Scale (NIHSS) score, and Barthel Index.

#### Stroke functional exercise adherence questionnaire scale (EAQ)

Consisting of 3 dimensions and 14 items (21), this assessment tool evaluates physical engagement in exercise, monitoring of exercise outcomes, and seeking advice. Each item is rated on a 4-point Likert scale ranging from 1 to 4, with higher cumulative scores indicating greater adherence. The adherence rate is calculated as follows: (adherence score/total number of items) × 100%. Adherence levels are classified into three categories: high (> 75%), medium (75 to 50%), and low (< 50%). The Cronbach’s *α* coefficient for this tool is 0.938.

#### Index of subjective well-being scale (IWB)

Originally developed by American psychologists Campbell et al. in 1976, the Chinese translation was conducted by Xiaodong Fan and consists of two parts assessed using a 7-point Likert scale. This tool has undergone extensive empirical research to establish its psychometric properties and is widely utilized (22). The total score is derived from the mean score of the overall affective index scale (consisting of 8 items) combined with the score from the life satisfaction questionnaire (a single item weighted at 1.1). Levels of happiness are categorized as follows: low happiness ranges from 2.1 to 6.0 points, medium happiness from 6.1 to 10.0 points, and high happiness from 10.1 to 14.7 points. The Cronbach’s *α* coefficient for this tool was 0.945.

#### Hospital anxiety and depression scale (HAD)

This self-assessment scale is utilized to identify and evaluate levels of anxiety and depression among patients in general hospital settings. Comprising two parts, namely the anxiety subscale (HAD-a) and the depression subscale (HAD-d), each section consists of 7 items rated on a 4-point scale (0, 1, 2, 3). Scores on the subscale are interpreted as follows: 0–7 indicating no symptom manifestation, 8–10 suggesting mild symptoms, and 11–21 indicative of significant symptoms (23). The Cronbach’s ɑ coefficient for this tool is 0.864.

### Data collection

The principal investigator and an assistant, one for each task, were primarily tasked with the distribution and collection of questionnaires. Before commencing the survey, a standardized explanation was provided outlining the survey’s objectives and guidelines for completing the questionnaires. All questionnaires were anonymous, gathered on-site upon completion, and promptly reviewed for any missing information.

### Data analysis

The data were statistically analyzed using SPSS 26.0, with an Excel database used for data entry. Descriptive statistics were employed, presenting measurement data as mean ± standard deviation and comparing them using *t*-tests or ANOVA. Categorical data were expressed as frequency and percentage. Single-factor analysis utilized rank sum tests. The correlation between adherence to rehabilitation exercises and subjective well-being, anxiety, and depression was evaluated using Pearson’s correlation coefficient. To investigate the factors influencing the adherence to rehabilitation exercise of patients with stroke, multiple linear regression models were used, with the total EAQ score serving as the dependent variable and variables that demonstrated statistical significance in univariate and correlation analysis as independent variables. The correlation between the identified influencing factors and adherence to rehabilitation exercise was analyzed through box plots and scatter fit plots derived from regression analysis. Statistical significance was established at *p* < 0.05.

### Ethical considerations

This study was approved by the Institutional Ethics Committee of the First Affiliated Hospital of Chongqing Medical University (Approval No. K2023-668). All participants in this study were early stroke patients with normal consciousness and cognitive ability, and all subjects provided informed consent and signed a written informed consent form. In addition, all methods were performed in accordance with the relevant guidelines and regulations.

## Results

### General information and *t*-test analysis of adherence to rehabilitation exercises in acute stroke patients

To investigate the factors influencing adherence to rehabilitation exercises in acute stroke patients, a total of 240 questionnaires were distributed, and 227 were effectively analyzed, yielding an effective recovery rate of 94.6%. The 227 participants included 144 male participants (63.4%) and 83 female participants (36.6%). Adherence to rehabilitation exercises varied based on age, education level, mRS, NIHSS score, Barthel Index, presence of ICU stay, and dysarticulation. These differences were statistically significant (*p* < 0.05), as shown in Table 1.

**Table 1:** General information and *t*-test analysis of adherence to rehabilitation exercise (*n* = 227).

Projects	Number of cases (%)	Adherence			Test statistic	*p*
		Low	Medium	High		
Gender					−0.255	0.79
Male	144 (63.4%)	25 (17.4%)	61 (42.3%)	58 (40.3%)		
Female	83 (36.6%)	14 (16.9%)	34 (41.0%)	35 (42.1%)		
Age (years)					9.572	<0.0001
18–59	79 (34.8%)	8 (10.1%)	28 (35.4%)	43 (54.5%)		
60–74	95 (41.9%)	15 (15.8%)	42 (44.2%)	38 (40%)		
≥75	53 (23.3%)	16 (30.2%)	25 (47.2%)	12 (22.6%)		
Social support					−1.959	0.05
Yes	215 (94.7%)	37 (17.2%)	92 (42.8%)	86 (40%)		
No	12 (5.3%)	2 (16.7%)	3 (25%)	7 (58.3%)		
Insurance type					2.966	0.05
Urban insurance	106 (46.7%)	15 (14.1%)	41 (38.7%)	50 (47.2%)		
Resident insurance	90 (39.6%)	17 (18.9%)	38 (42.2%)	35 (38.9%)		
Self-funded	31 (13.7%)	7 (22.6%)	16 (51.6%)	8 (25.8%)		
Education level					6.037	0.003
Primary and middle school	154 (67.8%)	28 (18.2%)	75 (48.7%)	51 (33.1%)		
High School	39 (17.2%)	6 (15.4%)	10 (25.6%)	23 (59.0%)		
University	34 (15.0%)	5 (14.7%)	10 (29.4%)	19 (55.9%)		
Monthly income (¥)					0.478	0.62
<1,500	38 (16.8%)	7 (18.4%)	19 (50%)	12 (31.6%)		
1,500–3,000	65 (28.6%)	10 (15.4%)	30 (46.1%)	25 (38.5%)		
>3,000	124 (54.6%)	22 (17.7%)	46 (37.1%)	56 (45.2%)		
Marital status					1.204	0.30
Married	208 (91.6%)	34 (16.3%)	90 (43.3%)	84 (40.4%)		
Unmarried	3 (1.3%)	1 (33.3%)	1 (33.3%)	1 (33.3%)		
Divorcee	16 (7.1%)	4 (25.0%)	4 (25.0%)	8 (50.0%)		
Presence of ICU stay					−3.093	0.002
Yes	48 (21.1%)	13 (27.1%)	19 (39.6%)	16 (33.3%)		
No	179 (78.9%)	26 (14.5%)	76 (42.5%)	77 (43.0%)		
Dysarticulation					2.079	0.03
No	137 (60.4%)	18 (13.1%)	57 (41.6%)	62 (45.3%)		
Mild and moderate	90 (39.6%)	21 (23.3%)	38 (42.2%)	31 (34.5%)		
mRS					11.649	<0.0001
Level 2	148 (65.2%)	15 (10.1%)	62 (41.2%)	71 (47.8%)		
Level 3	15 (6.6%)	4 (26.7%)	7 (46.6%)	4 (26.7%)		
Level 4	64 (28.2%)	20 (31.3%)	26 (40.6%)	18 (28.1%)		
NIHSS score					16.949	<0.0001
Mild	170 (74.9%)	17 (10.0%)	70 (41.2%)	83 (48.8%)		
Moderate	54 (23.8%)	20 (37.0%)	24 (44.5%)	10 (18.5%)		
Moderate–severe	3 (1.3%)	2 (66.7%)	1 (33.3%)	0 (0.0%)		
Barthel Index					4.153	0.04
Mild (61 ~ 99)	169 (74.4%)	27 (16.0%)	69 (40.8%)	73 (43.2%)		
Moderate (41 ~ 60)	58 (25.6%)	12 (20.7%)	26 (44.8%)	20 (34.5%)		
Severe (≦40分)	0 (0.0%)	0 (0.0%)	0 (0.0%)	0 (0.0%)		

### Scores of EAQ, IWB, and HAD in acute stroke patients

The mean adherence rate was 68.6%. The scores of other dimensions among stroke patients are shown in Table 2.

**Table 2:** Scores on the EAQ, IWB, and HAD (*n* = 227).

Scale	Projects	Items	Mean scores	Classification [cases (%)]			
				Low	Medium	High	Mean adherence (%)
EAQ	Total score	14	38.41 ± 11.13	39 (17.2%)	95 (41.8%)	93 (41.0%)	68.6
	Dimension 1	8	22.89 ± 7.05	41 (18.1%)	79 (34.8%)	107 (47.1%)	71.5
	Dimension 2	3	7.93 ± 2.48	34 (15.0%)	135 (59.5%)	58 (25.5%)	66.1
	Dimension 3	3	7.59 ± 2.96	59 (26.0%)	102 (44.9%)	66 (29.1%)	63.3
IWB	Total score	9	11.20 ± 2.54	5 (2.2%)	70 (30.8%)	152 (67.0%)	
	Emotional index	8	5.16 ± 1.29				
	Life satisfaction	1	6.03 ± 1.45				
				*Negative*	*Suspicious*	*Positive*	
HAD	HAD(a)	7	4.57 ± 3.36	178 (78.4%)	39 (17.2%)	10 (4.4%)	
	HAD(d)	7	6.15 ± 4.34	147 (64.8%)	40 (17.6%)	40 (17.6%)	

*Adherence: Low<50%, Medium 50–75%, High>75%; Well-being: Low 2.1–6.0 points, Medium 6.1 to 10.0 points, High 10.1–14.7 points; Anxiety or Depressive: Negative<8 points, Suspicious 8–10 points, Positive >10 points. Dimension 1: Exercise adherence, Dimension 2: Exercise monitoring adherence, Dimension 3: Active advice Seeking adherence.*

### Correlation analysis of EAQ with IWB and HAD

Pearson correlation analysis revealed a positive correlation between adherence to rehabilitation exercises and subjective well-being (*r* = 0.526, *p* < 0.01), along with negative correlations with anxiety (*r* = −0.429, *p* < 0.01) and depression (*r* = −0.291, *p* < 0.01), as presented in Table 3.

**Table 3:** Correlation analysis of EAQ with IWB and HAD.

Projects	EAQ	Dimension 1	Dimension 2	Dimension 3	IWB	HAD	HAD(a)	HAD(d)
EAQ	1							
Dimension 1	0.941**	1						
Dimension 2	0.890**	0.737**	1					
Dimension 3	0.774**	0.537**	0.751**	1				
IWB	0.526**	0.476**	0.482**	0.439**	1			
HAD	−0.429**	−0.411**	−0.380**	−0.315**	−0.632**	1		
HAD(a)	−0.291**	−0.282**	−0.245**	−0.218**	−0.490**	0.887**	1	
HAD(d)	−0.470**	−0.447**	−0.427**	−0.343**	−0.645**	0.934**	0.664**	1

**Significant correlation at the 0.01 level (two-tailed).

### Results of multifactorial analysis of adherence to rehabilitation exercise

An ordered logistic regression analysis was conducted using the EAQ score as the dependent variable and key variables identified as statistically significant in the univariate analysis—age, education level, mRS, NIHSS score, Barthel Index, presence of ICU stay, dysarticulation, as well as total scores from HAD and IWB—as independent variables. The findings indicated that age (*p* < 0.0001), presence of ICU stay (*p* = 0.03), NIHSS score (*p* < 0.0001), and subjective well-being (*p* < 0.0001) were significant factors influencing adherence to rehabilitation exercises in early stroke patients, as illustrated in Table 4.

**Table 4:** Multifactorial analysis of adherence to rehabilitation exercise.

Independent variable	*β*	Standard error	*t*	*p*	95.0% CI lower limit	95.0% CI ceiling
(Constant)	30.833	7.803	3.951	<0.0001	15.453	46.213
Age (years)	−3.225	0.762	−4.23	<0.0001	−4.727	−1.722
Education level	0.903	0.795	1.136	0.25	−0.664	2.47
Presence of ICU stay	3.258	1.446	2.253	0.02	0.408	6.107
Dysarticulation	−1.002	1.198	−0.837	0.40	−3.364	1.359
mRS	−0.604	0.622	−0.972	0.33	−1.83	0.621
NIHSS score	−5.087	1.52	−3.348	<0.0001	−8.083	−2.092
Barthel index	0.898	1.367	0.657	0.51	−1.797	3.593
IWB	1.621	0.293	5.535	<0.0001	1.044	2.199
HAD	−0.155	0.106	−1.463	0.14	−0.364	0.054

### Effect of the identified influencing factors on adherence to rehabilitation exercise

**Figure 2:**
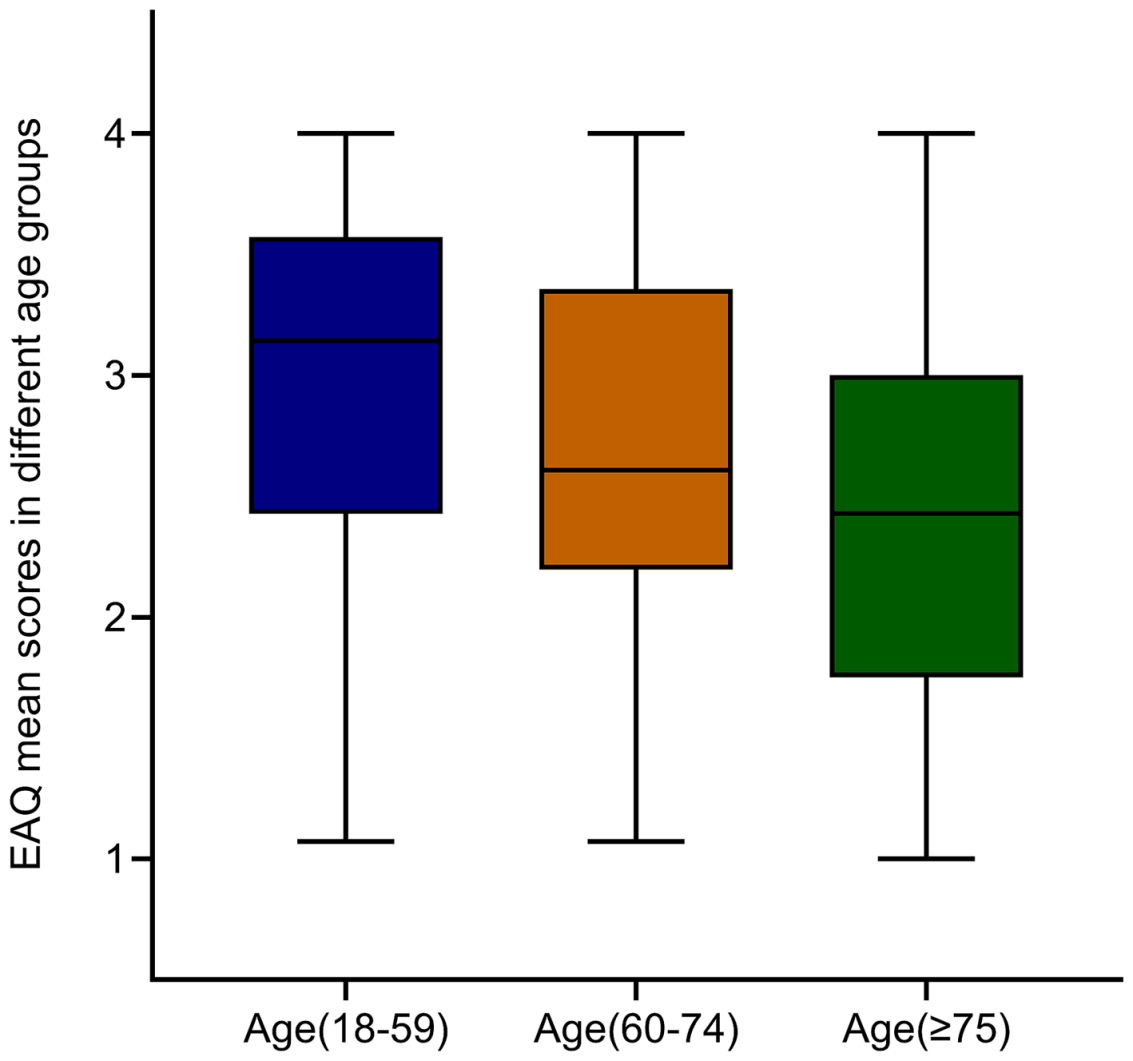
EAQ mean scores in different age groups. Decreased adherence to rehabilitation exercise with increasing age. Data are mean ± SEM; *p*-values were calculated using one simple *t* test, *p* < 0.001.

**Figure 3:**
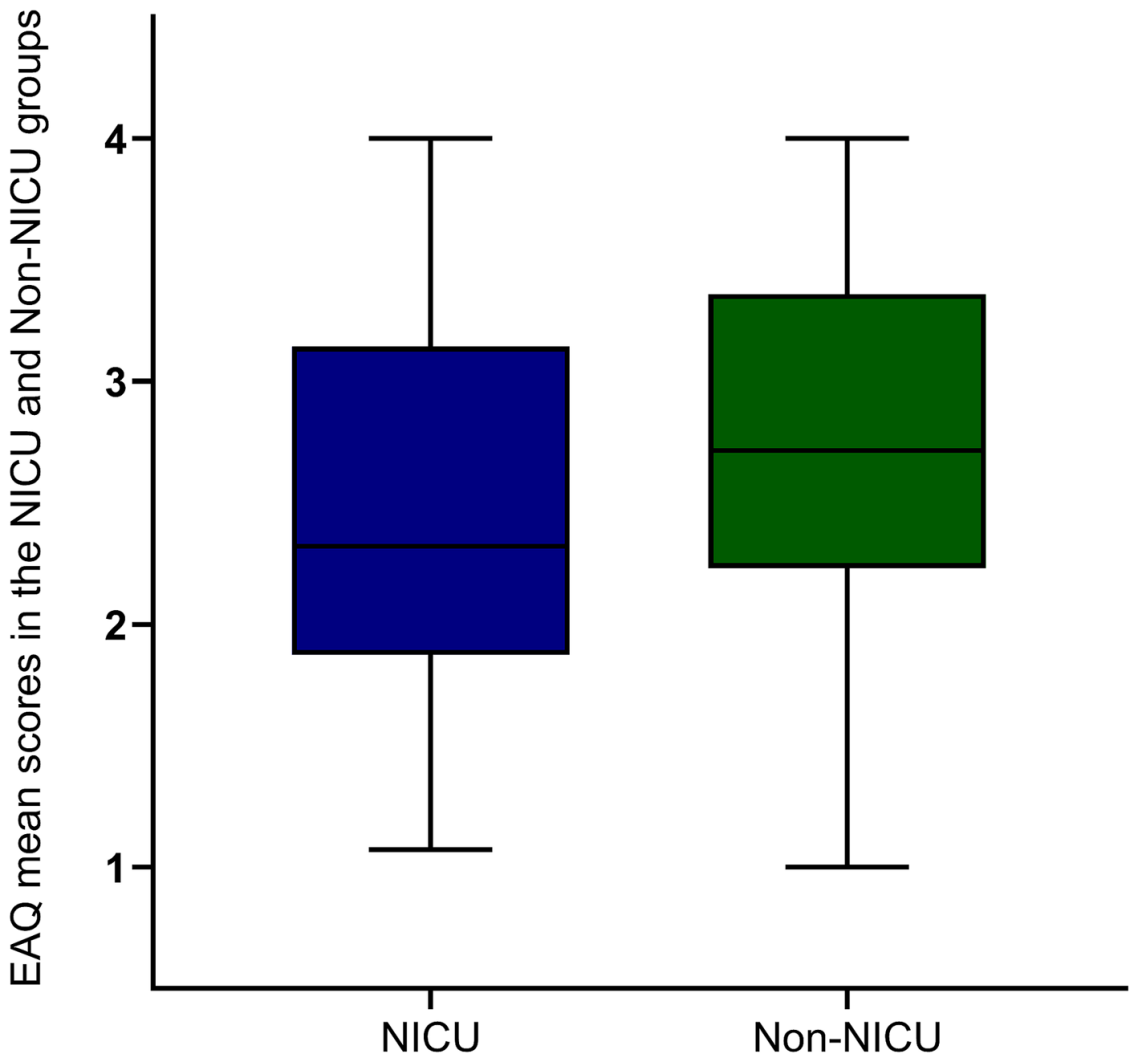
EAQ mean scores in the NICU and non-NICU groups. NICU has lower adherence to rehabilitation exercises than non-NICU. Blue and green represent NICU and non-NICU. Data are mean ± SEM; *p*-values were calculated using one simple *t* test, *p* < 0.001.

**Figure 4:**
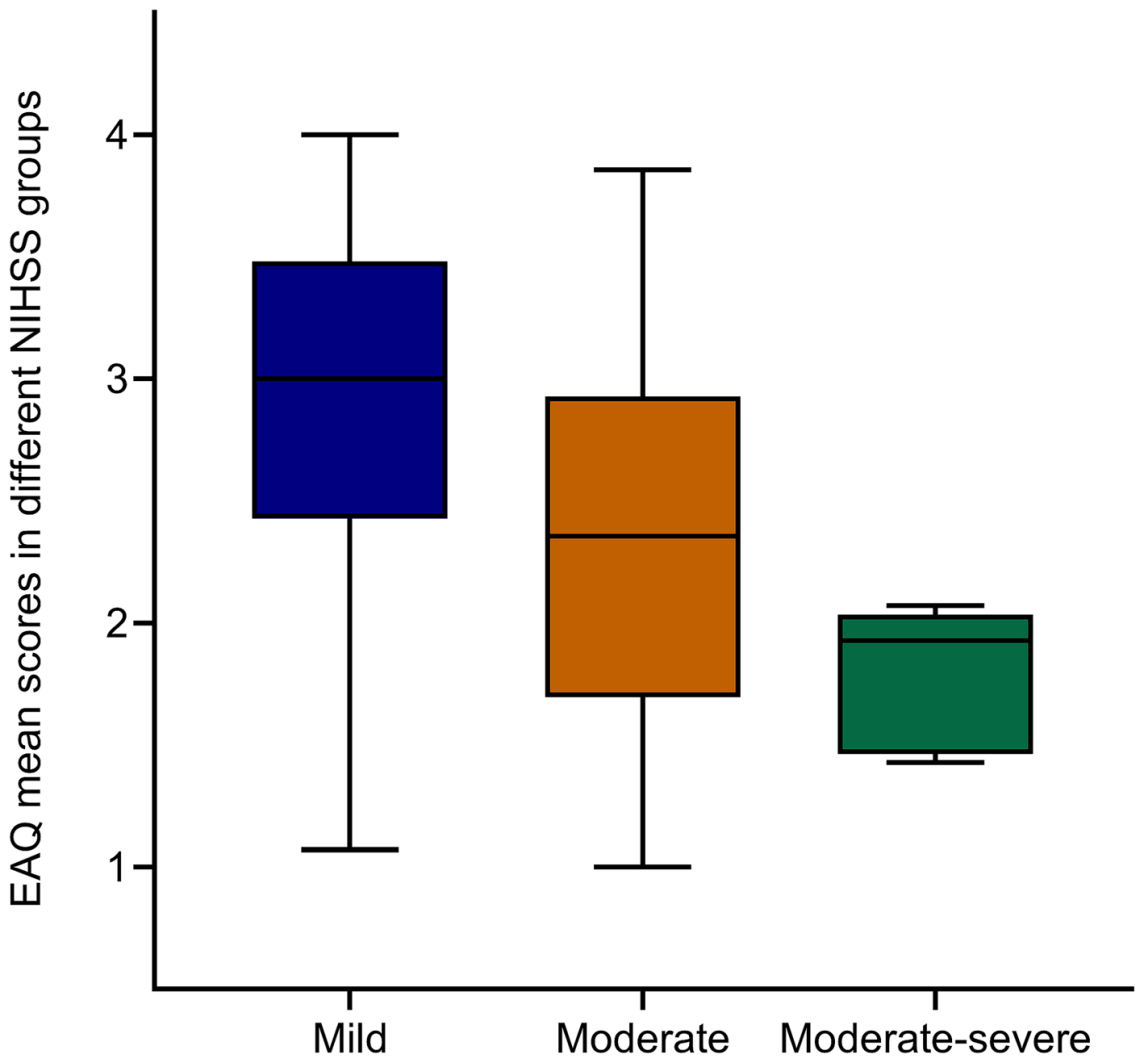
EAQ mean scores in different NIHSS groups. Decreased adherence to rehabilitation exercise with increasing NIHSS score. Data are mean ± SEM; *p*-values were calculated using one simple t test, *p* < 0.005.

**Figure 5:**
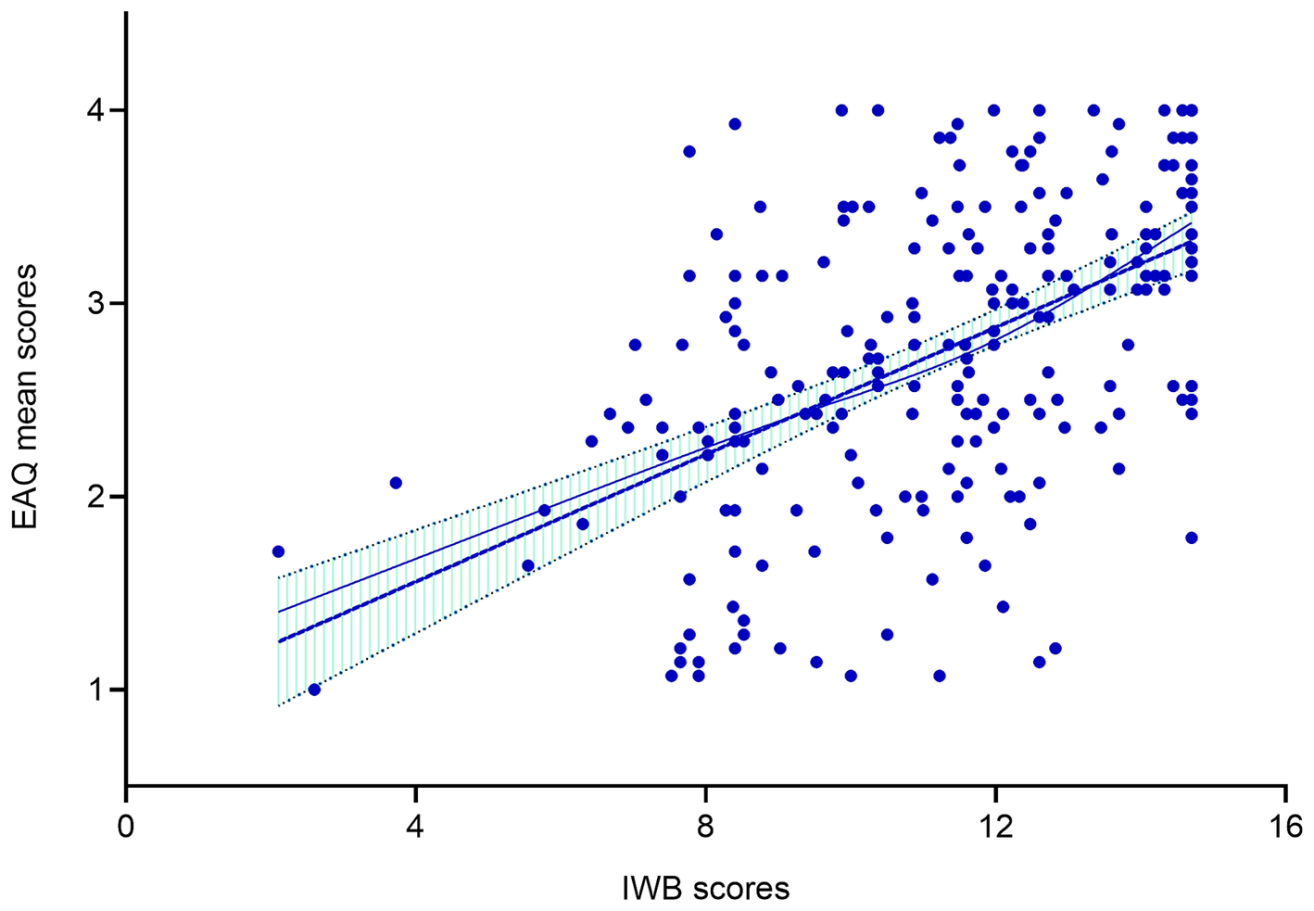
Regression analysis of subjective well-being and rehabilitation exercise adherence. Increased adherence to rehabilitation exercise with increasing subjective well-being. Blue points represent EAQ mean scores; Blue line represent fitted value; Dotted green area represent 95% CI; *p*-values were calculated using simple linear regression, *p* < 0.001.

The results showed that age, presence of ICU stay, and NIHSS score had significant effects on adherence to rehabilitation exercise. These variables were visually represented using box plots as shown in Figures 2–4. The relationship between subjective well-being and adherence to rehabilitative exercise was observed using scatter fit plots derived from regression analysis, as shown in Figure 5.

## Discussion

### The rehabilitation exercise adherence in acute stroke patients is medium

The current study employed a cross-sectional survey to examine the status and influencing factors of rehabilitation exercise adherence among individuals with acute stroke. The findings revealed that the mean total score for rehabilitation exercise adherence in acute stroke patients was 38.41 ± 11.13, with an average adherence rate of 68.6%, which is considered intermediate. Only 17.2% of patients had low adherence (<50%), with the majority falling within the medium to high adherence range. These results surpassed the adherence rates reported in other studies: Odetunde, M.O. et al. (24) reported that 33.3% of patients had poor exercise adherence, Miller et al. (25) indicated that patient adherence to home exercise programs (HEP) after discharge from rehabilitation was less than ideal, and Li et al. (26) reported an adherence rate of 53.89 ± 6.28%. Conversely, the results of this study were lower than those reported by Zhang et al. (27), who found a total score of functional exercise adherence in young and middle-aged hemorrhagic stroke patients to be 43.18 ± 9.57, with an adherence rate of 77.11%, indicating a high level of compliance. This discrepancy may be attributed to the higher proportion of patients with mild to moderate neurological deficits in the hospitalized group who received comprehensive health education on rehabilitation through training videos, which reinforced the importance of rehabilitation and increased adherence to prescribed exercises. Notably, the study found that adherence rates among young and middle-aged patients were around 54.5%. Younger patients often have a greater sense of urgency to reintegrate into their families and communities, leading to higher adherence rates to rehabilitation exercises.

### Age, presence of ICU stay, and NIHSS score have negative correlations with rehabilitation exercise adherence

In this study, age, education level, mRS, Barthel Index, NIHSS score, presence of ICU stay, and dysarticulation significantly influenced patients’ adherence to rehabilitation exercises (*p* < 0.05). Gender was not a factor in adherence; the sample included 144 male participants (63.4%) and 83 female participants (36.6%). The lack of statistical difference in *p*-values between males and females may be due to the sample size or distribution. Similar to previous studies, age (28, 29) and NIHSS (30–32) negatively correlate with adherence to rehabilitative exercise: the older the age, the lower the adherence; the higher the NIHSS, the lower the adherence. The occurrence of concomitant aphasia during the acute phase of stroke ranges from 21 to 38% (33). Our study found that patients with mild to moderate dysarticulation had lower adherence to rehabilitation exercises than those without speech impairment. This is in line with a study (26) that showed stroke patients with coexisting speech disorders had even lower adherence to rehabilitation exercises. Such challenges may be due to communication barriers that hinder problem-solving during exercises, leading to reduced adherence. Additionally, patients with speech disorders may experience increased negative emotions and decreased motivation to engage in exercise following a stroke. Some studies suggest that one in four post-stroke patients eventually becomes depressed, with aphasia being identified as a major risk factor (34). The reported prevalence is approximately 52% (35). Furthermore, the presence of an ICU transfer was negatively associated with adherence to rehabilitation exercises. Patients transferred from the Intensive Care Unit (ICU) have lower adherence to rehabilitation exercises than those not transferred. Potential reasons for this lower adherence may include: firstly, the severity of the patients’ condition and concerns about disease prognosis; secondly, transfer from a familiar to an unfamiliar environment may induce migratory stress, leading to increased levels of anxiety, depression, and feelings of loneliness (36). The above results showed that negative psychological factors were negatively correlated with adherence to rehabilitation exercises in stroke patients.

### Subjective well-being have positive correlation with rehabilitation exercise adherence

Interestingly, subjective well-being is positively associated with adherence to rehabilitation exercises. In our study, we observed that the mean subjective well-being score among post-stroke patients was 11.20 ± 2.54. This is similar to the results of the Campbell study conducted by Campbel in 1971, in which 2,160 adults over the age of 18 were assessed using the Index of Happiness scale. The study reported a mean score of 11.8 (standard deviation of 2.2) for the whole sample. The reason for the similarity is that our study focused on acute stroke patients who were coping with the challenges of the disease, and stroke survivors showed post-traumatic growth. Qualitative analysis showed that the most common positive changes were a greater appreciation of life and more intense/selective relationships. Considering positive changes may provide an additional perspective for recovery (37). Published models linking positive emotions to cardiac outcomes include both behavioral and physiological components (38). Positive emotions such as happiness appear to be associated with increased participation in heart-healthy behaviors (e.g., healthy eating, physical activity) that are associated with beneficial outcomes (39, 40). Data linking positive mental states to biomarkers of heart health, such as markers of inflammation, are mixed but suggest a potential association (41). Although no existing studies have directly correlated subjective well-being with adherence to rehabilitation exercises in acute stroke patients, studies have explored the impact of positive psychological interventions on subjective well-being after acquired brain injury. For example, a 2016 study highlighted the prevalence of psychological distress following ABI and the limited evidence for psychotherapeutic interventions (42). However, improvements in subjective well-being, anxiety, and depression have been reported with such interventions. Recent studies by Lambiase et al. (43), Wang X. et al. (44), and Zheng et al. (45) adopted positive psychological interventions in the care of stroke patients and demonstrated positive outcomes in alleviating negative emotions and improving subjective well-being. Therefore, healthcare providers should consider implementing positive psychological interventions as part of patient care and continue them for a long time, not only to address existing challenges and alleviate negative emotions but primarily to promote positive emotions, cognition, and behavior. This holistic approach aims to increase subjective well-being and improve adherence to rehabilitation in stroke patients.

### Limitations

This study has limitations: (1) This study was limited to a single-center quantitative cross-sectional analysis, which may affect the generalizability of the results. (2) The NIHSS and mRS scores of the study subjects were predominantly mild, with fewer subjects having moderate and severe scores. (3) General information was missing items such as geography.

## Conclusion

The study found that the overall level of adherence to rehabilitation exercises in acute stroke patients was medium and influenced by several factors. In particular, younger age, non-ICU transfer status, lower NIHSS scores, and higher subjective well-being were associated with increased adherence to exercise rehabilitation in this patient population. Future research will include multi-center studies with qualitative interviews of stroke patients to gain a deeper understanding of the factors that promote or hinder rehabilitation exercise. I am working to compare and contrast these factors, but this requires comprehensive improvement. Based on the identified factors, a positive psychological intervention program will be developed to test its effects on improving patients’ adherence to rehabilitation exercises.

## Data Availability

The original contributions presented in the study are included in the article/supplementary material, further inquiries can be directed to the corresponding authors.
